# Hyperspectral analysis applied to micro-Brillouin maps of amyloid-beta plaques in Alzheimer's disease brains

**DOI:** 10.1039/c8an01291a

**Published:** 2018-11-21

**Authors:** Francesca Palombo, Francesco Masia, Sara Mattana, Francesco Tamagnini, Paola Borri, Wolfgang Langbein, Daniele Fioretto

**Affiliations:** a University of Exeter , School of Physics and Astronomy , Exeter EX4 4QL , UK . Email: f.palombo@exeter.ac.uk; b Cardiff University , School of Physics and Astronomy , Cardiff CF24 3AA , UK; c CNR – National Institute of Optics , Firenze I-50125 , Italy; d University of Reading , School of Pharmacy , Reading RG6 6LA , UK; e Cardiff University , School of Biosciences , Cardiff CF10 3AX , UK; f University of Perugia , Department of Physics and Geology , Perugia I-06100 , Italy

## Abstract

A recent investigation on the architecture and chemical composition of amyloid-β (Aβ) plaques in *ex vivo* histological sections of an Aβ-overexpressing transgenic mouse hippocampus has shed light on the infrared light signature of cell-activation related biomarkers of Alzheimer's disease. A correlation was highlighted between the biomechanical properties detected by Brillouin microscopy and the molecular make-up of Aβ plaques provided by FTIR spectroscopic imaging and Raman microscopy (with correlative immunofluorescence imaging) in this animal model of the disease. In the Brillouin spectra of heterogeneous materials such as biomedical samples, peaks are likely the result of multiple contributions, more or less overlaid on a spatial and spectral scale. The ability to disentangle these contributions is very important as it may give access to discrete components that would otherwise be buried within the Brillouin peak envelope. Here, we applied an unsupervised non-negative matrix factorization method to analyse the spontaneous Brillouin microscopy maps of Aβ plaques in transgenic mouse hippocampal sections. The method has already been proven successful in decomposing chemical images and is applied here for the first time to acoustic maps acquired with a Fabry–Perot Brillouin microscope. We extracted and visualised a decrease in tissue rigidity from the core through to the periphery of the plaque, with spatially distinct components that we assigned to specific entities. This work demonstrates that it is possible to reveal the structure and mechanical properties of Aβ plaques, with details visualized by the projection of the mechanical contrast into a few relevant channels.

## Abstract 2

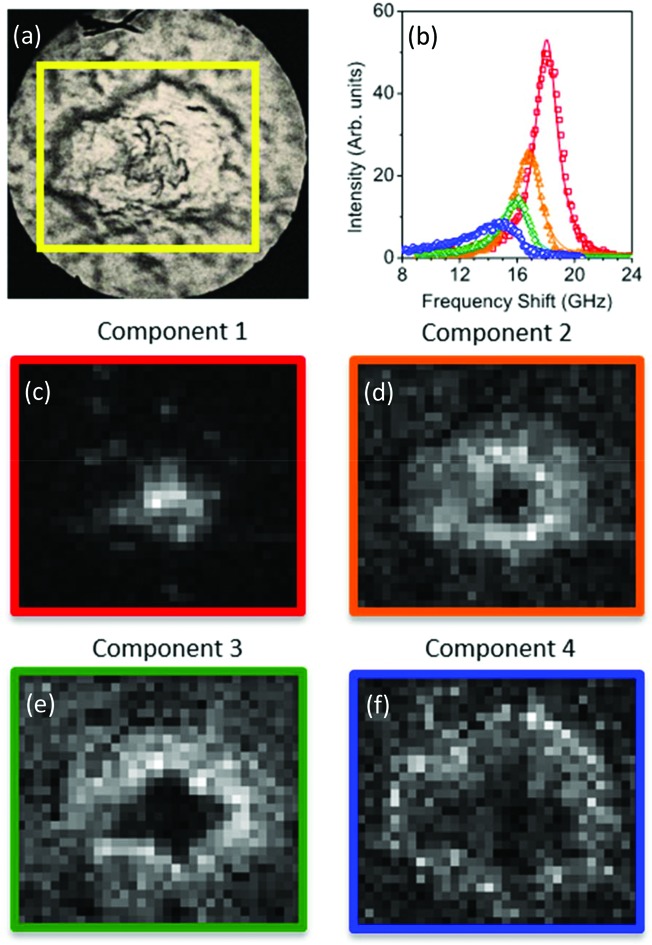
Non-negative factorization analysis applied to spontaneous Brillouin microscopy maps of amyloid-beta plaques in a transgenic mouse model enables to resolve spatially distinct components with specific mechanical properties.

## Introduction

Dementia is a prevalent disease, which affects around 47 million people worldwide, a number that is predicted to triple by 2050 based on current trends due to an ageing population.^1^ Amyloidopathy is a major hallmark of Alzheimer's disease (the most common form of dementia) and consists of abnormal deposits of the amyloid-β (Aβ) peptide in the brain parenchyma.

There is no reliable and affordable diagnostic tool for Alzheimer's disease *in vivo*. This has prompted early applications of vibrational spectroscopy and imaging techniques to investigate the structure and molecular constituents of Aβ plaques in the brain of both humans and mouse models of the disease.^2^,^3^ These studies have revealed the presence in many plaques of a lipid-rich layer surrounding a core that is made of intermolecular β-sheet structures of the Aβ peptide. We clarified the origin of this lipid layer in senile plaques within *ex vivo* hippocampal sections of Aβ-overexpressing TASTPM mouse brain (a model for familial Alzheimer's disease) using a combination of chemical imaging (FTIR spectroscopic imaging and Raman microscopy) and immunofluorescence imaging.^4^ The lipid layer stained positive for astrocytes co-localized with the lipid-loaded ring. This was an important result as it shows that FTIR and Raman techniques not only are sensitive to the Aβ peptide structure and aggregation, but they can also detect signatures of astrogliosis and possibly other biomarkers of inflammation with potential for clinical translation.

Brillouin spectroscopy is an emerging biophotonic technique, which probes the micromechanical properties of matter through the frequency shift of light scattered off thermally induced acoustic waves or *phonons* at high frequency (GHz). It is an optical, contactless and non-destructive technique, and probes native signals that relate to the viscoelasticity or stiffness of matter on a micro-scale. Typically a Brillouin spectrum contains one or more sets of symmetric inelastic scattering peaks to the central Rayleigh line; these peaks arise from Doppler-shifted longitudinal phonons propagating with a frequency equal to the Brillouin shift.^5^ We applied Brillouin microscopy to a range of biological and medical samples including protein fibres,^6^–^8^ biofilms,^9^,^10^ live cells,^11^ normal tissues^6^ and pathologies including pre-cancerous stages in epithelial tissue^12^,^13^ and Aβ plaques in an Alzheimer's brain.^14^ We found important correlations between chemical composition, structure and micromechanics, which form the basis for further investigations on living systems and translation to the clinics. For the first time, the micromechanical properties of the lipid membrane in astrocyte processes surrounding Aβ plaque cores in *ex vivo* sections of transgenic mouse hippocampus were characterised using Brillouin scattering.^14^

As for other spectroscopic techniques, the Brillouin peak has two major contributions to the observed linewidth: homogeneous broadening (due to local viscosity affecting each mode identically) and heterogeneous broadening (arising from mechanically heterogeneous ‘environments’ within the scattering volume). Multiple-scattering processes in turbid media may also result in effective broadening. However, due to the few micrometres of interaction length below the sample surface in the confocal Brillouin scattering setup used, multiple scattering can be neglected for the data presented. Additionally, asymmetric broadening is generated by the finite numerical aperture of collection of scattered light. In the present data, this effect is small (∼1%) due to the small NA of the objective used, and thus does not provide a significant broadening.^14^ The heterogeneous character of line broadening in Brillouin spectra is an issue that insofar has been solved only in the case of sufficiently separated contributions such as those from live cells and the buffer saline.^11^ For biomedical applications, this is a particularly pressing issue. In Aβ plaques for which we have demonstrated the mechanical heterogeneity in a mouse model of amyloidopathy,^4^ this is apparent as the Brillouin peak detected in the mapping mode from any spatial location at low magnification (20×) arises from multiple contributions from different molecular constituents. An in-depth investigation of the molecular species involved in the mechanism of Aβ plaque formation is the first step to unravel the pathophysiology of this complex disease.

The aim of this work was to investigate whether it is possible to separate multiple phonon modes contributing to the spectral band shape of the Brillouin peak in a heterogeneous sample. We used an Aβ-overexpressing transgenic mouse model of Alzheimer's disease, TASTPM mice that carry a double mutation on the amyloid precursor protein (APP) and one single mutation on the presenilin-1 gene causing prevalent amyloid plaque deposition before they are six months old.^15^,^16^ Aβ deposits within the histological sections of the fixed brain hippocampus from TASTPM mice were analysed. In this work, we tested a new method based on non-negative matrix factorization (NMF)^17^,^18^ to decompose Brillouin hyperspectral datasets of Aβ plaques in transgenic mice hippocampus. In this method, the hyperspectral images are interpreted as a linear combination of a limited number of components, each characterised by a spectrum and a concentration map. Both spectra and spatial maps have a non-negativity constraint, hence the basis is not orthonormal. The algorithm searches for the set of spectra and spatial distributions which minimise the factorization error. Here we provide evidence that distinct spatial locations within Aβ plaques – which correspond to specific molecular species – have differential viscoelastic properties probed by Brillouin spectroscopy. This opens up new avenues for data analysis of micro-Brillouin images based on chemometrics and multivariate statistics.

## Results

**Fig. 1:**
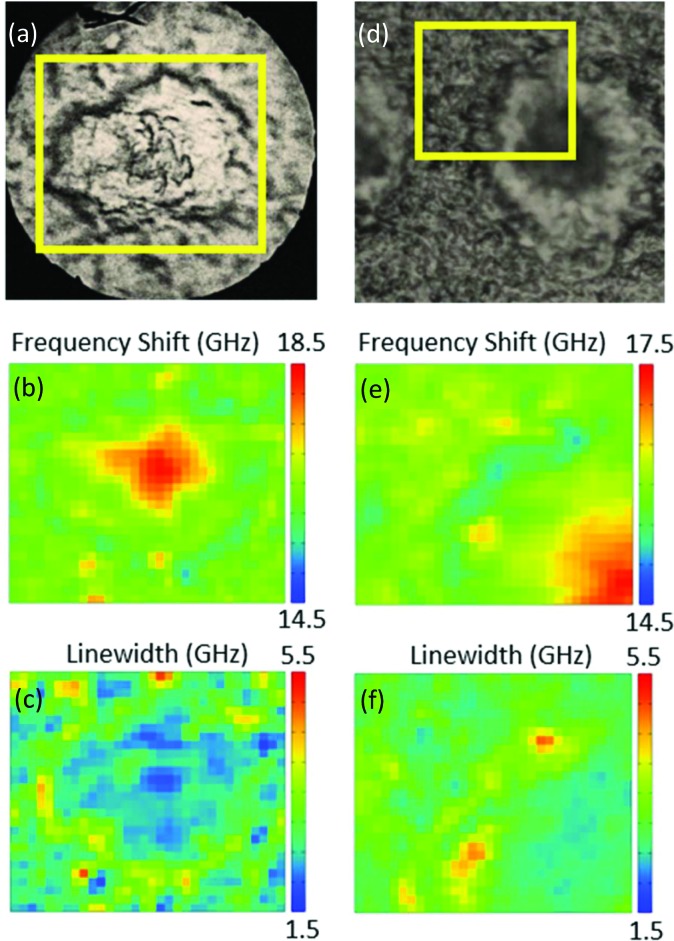
(Top panel) Reflection optical images of plaques in transgenic mouse brain sections containing the hippocampus. Yellow boxes indicate the 66 × 60 μm^2^ and 50 × 43 μm^2^ areas of the Brillouin map of (a) a whole plaque and (d) a quarter of a plaque. (Middle and bottom panels) Brillouin images refer to the distribution of (b, e) frequency shift and (c, f) linewidth derived from DHO fit analysis applied to the Brillouin peak across the map spectra (see Methods).

Fig. 1 shows bright-field reflection and Brillouin images of amyloid plaques in transgenic mouse brain sections, whereby the plaque core is easily recognizable from the elevated frequency of the Brillouin peak. This has previously been shown to correspond to the intermolecular β-sheets of the aggregated Aβ peptide detected by FTIR and Raman spectroscopy applied to the same tissue sections.^4^

Indeed, the elevated rigidity of the plaque core observed here can be attributed to the deposition of aggregates of β-amyloid protein in β-pleated sheet conformation and to the exclusion of hydration water from this highly hydrophobic region.^14^ A lipid-rich layer surrounding the plaque core was apparent in the chemical images as well as in the mechanical maps through a depleted frequency shift (Fig. 1b and e) and an increased linewidth (Fig. 1c and f) of the Brillouin peak.

**Fig. 2:**
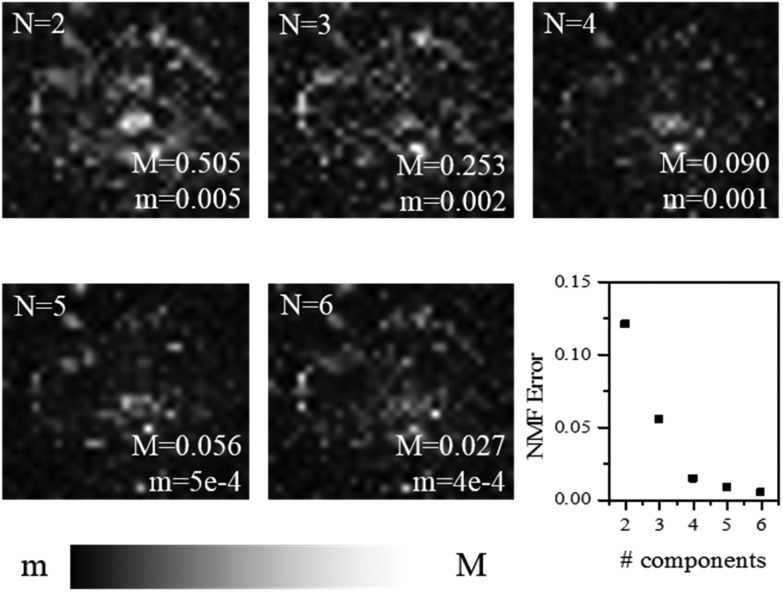
Factorization error of the NMF analysis applied to the map of a whole plaque (Fig. 1a) as a function of the number of components *N* used. Spatially resolved error maps for *N* = 2 to 6 on a greyscale as shown, and the global error, are given.

Moving on to the factorization analysis, we note that the only supervised parameter we have to choose is the number of components used. It was chosen here to result in a spectral error without an obvious remaining spatial structure (see Fig. 2, which refers to the map of a whole plaque shown in Fig. 1a).

**Fig. 3:**
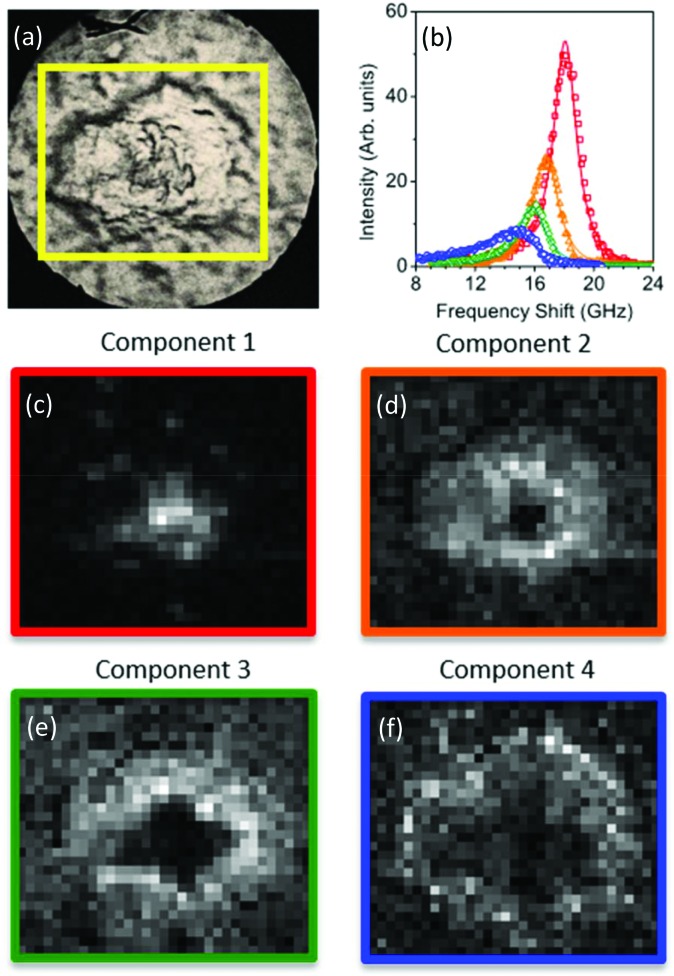
(Top panel) Reflection optical image of a plaque in a transgenic mouse brain section and factorisation basis spectra retrieved by NMF. (a) Photomicrograph of the plaque with a yellow box denoting the region of the Brillouin map. (b) Spectra of the four factorisation components. Symbols denote the data and lines indicate the DHO fit result. The components are colour coded so that (middle and bottom panels) the corresponding concentration maps are also presented. The four components can be attributed (see text) to the distribution of (c) the plaque core made of β-sheet structures, presenting a spectrum with the largest frequency shift, (d) a first intermediate region, (e) a second intermediate, more diffuse region, and (f) the periphery of the plaque having a broad linewidth and small frequency shift, plausibly related to the lipid-rich region of the astrocyte processes. The greyscale of the concentration maps goes from 0 (black) to 1 (white). The map corresponds to a 66 × 60 μm^2^ area of the specimen acquired with a step size of 1.5 μm.

**Fig. 4:**
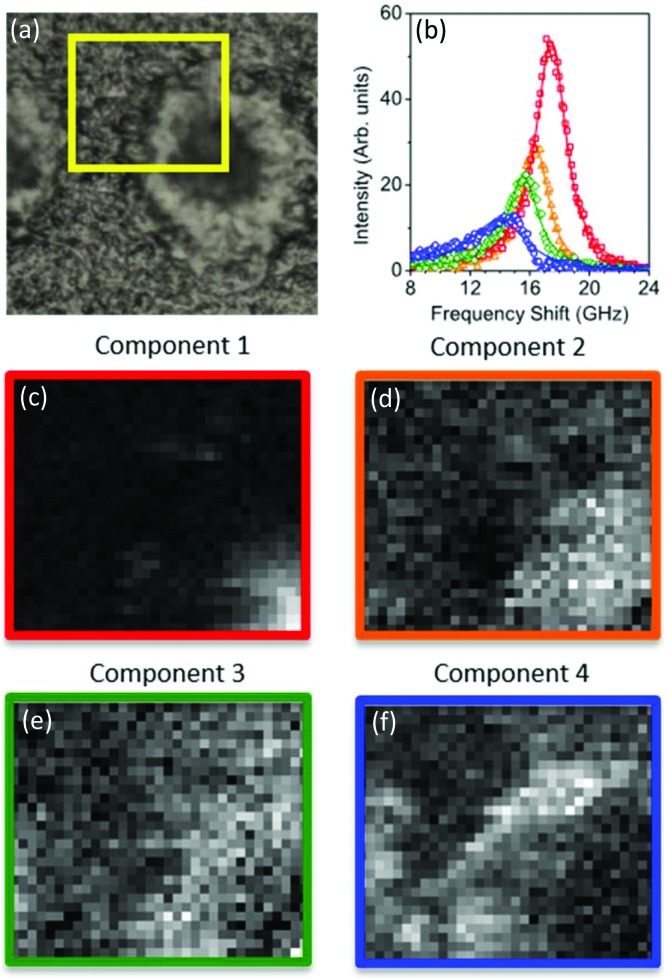
(Top panel) Reflection optical image of a quarter of a plaque in a transgenic mouse brain section and factorisation basis spectra retrieved by NMF. (a) Photomicrographs of the plaque with a yellow box denoting the region of the Brillouin map. (b) Spectra of the four factorisation components. Symbols denote the data and lines indicate the DHO fit result. The components are colour coded so that (middle and bottom panels) the corresponding concentration maps are also presented. The four components can be attributed (see text) to the distribution of (c) the plaque core made of β-sheet structures, presenting a spectrum with the largest frequency shift, (d) a first intermediate region, (e) a second intermediate, more diffuse region, and (f) the periphery of the plaque having a broad linewidth and small frequency shift, plausibly related to the lipid-rich region of the astrocyte processes. The greyscale of the concentration maps goes from 0 (black) to 1 (white). The map corresponds to a 50 × 43 μm^2^ area of the specimen acquired with a step size of 1.5 μm.

The spectral error at each position is defined as the root-mean-square of the error over the spectral points normalized by the Frobenius norm of the measured data over these points.^17^ For 2 and 3 components, the spectral error shows significant spatial structures, both at the core and at the edge of the plaque, indicating that there are too few components to describe the data within its noise. By using 4 or more components, no clear spatial structure in the error is observed. Furthermore, the global error, defined as the ratio of the Frobenius norms of the error and the data over all data, decreases with increasing number of components (see Fig. 2) and shows a significantly lower decrease adding a fifth component compared to adding the third or fourth component. Moreover, when using more than 4 components, the factorization returns pairs of components which have similar spatial distributions, indicating that the algorithm is splitting a physically meaningful component into two. We therefore use in the following 4 components, for which the results of the unsupervised decomposition method are reported in Fig. 3 and 4.

The linewidths of the components resolved by the factorization analysis are less than 5 GHz (see below), hence they are smaller than the linewidths previously obtained by fitting each spectrum of the maps to a single damped harmonic oscillator (DHO) function (Fig. 1c and f). This suggests that the partially heterogeneous nature of the Brillouin peaks can be resolved by the hyperspectral analysis, giving a reasonable proxy for the different biological components of the tissue.

When comparing these results with those from the analogous decomposition of vibrational spectroscopic images (*e.g.* from IR and Raman microscopy techniques), we must note that Brillouin microscopy cannot access detailed molecular level information since it reveals collective excitations (acoustic phonons) with frequency and linewidth given by average mechanical properties over micrometric mean free paths. However, heterogeneity in biological matter is expressed at various length scales, and it is invaluable to be able to resolve mechanical heterogeneities on a micrometre scale within the scattering volume. The unsupervised factorization method applied here is the first step in this direction, as demonstrated by these results, which are consistent with the biochemical composition of the plaques in terms of lineshape and intensity distribution of the components.

**Fig. 5:**
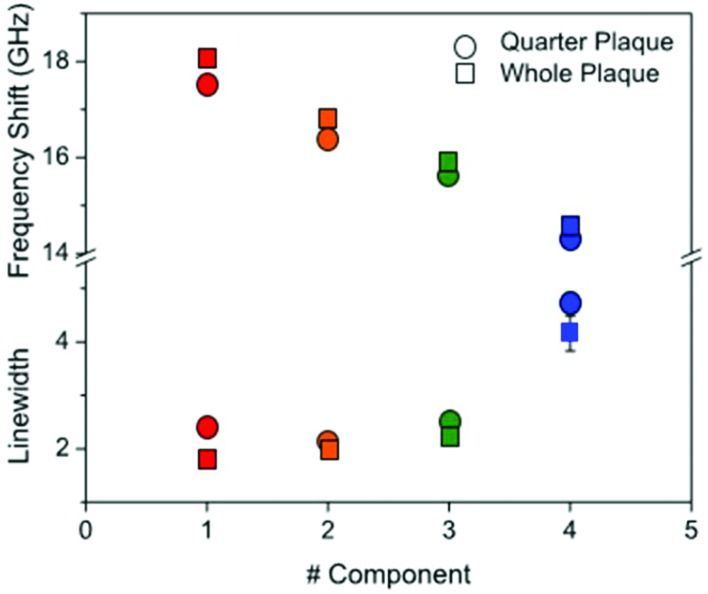
Plot of (top part) the frequency shift and (bottom part) linewidth of the four components derived from NMF applied to the Brillouin maps of Aβ plaques in *ex vivo* sections of transgenic mouse hippocampus. Symbols are colour coded according to the components as shown in Fig. 3 and 4. The components were fitted to a DHO function and the resulting frequency and linewidth are shown.

For each of the two spectral datasets (Fig. 3 and 4), the lineshape of the four components was fitted to a homogeneous model, described by a DHO function; results in terms of peak position and linewidth are shown in Fig. 5. It can be seen that, in all cases, the homogeneous model gives a reasonably good fit. The quality of the fit deteriorates for components with spectra peaked at smaller frequencies, *i.e.* component 4. We attribute this disagreement to a partial heterogeneous broadening of the spectra which is not separated into different components by NMF. This indicates that the spatial maps do not show sufficient regions of the corresponding homogeneous constituents. Such limitations could be overcome if, within the NMF algorithm, the component spectra would be constrained to DHO model shapes, which is a promising avenue for future development of the algorithm.

With regard to the assignment of the concentration images (greyscale images in Fig. 3 and 4), a comparison with the biological composition of the tissue suggests that:

• Component 1 (Fig. 3c and 4c), the component with the largest frequency shift, corresponding to the largest stiffness, is essentially consistent with the plaque core, made of the aggregated Aβ peptide, a hydrophobic region from which water is excluded. The rigidity of this region can be explained in terms of the mechanical properties of dense core plaques.

• Component 2 (Fig. 3d and 4d) is mainly distributed around the core, in close proximity but yet in a complementary (spatially distinct) area. This can be identified as a transition region, plausibly including α-helical structures of the Aβ peptide, with different levels of aggregation, which form the interfacial region between the largely hydrophobic plaque core and the hydrophilic extracellular matrix (ECM) compartment. It is expected that the physical properties of the tissue are such that there is a gradient in solubility from the dense-core Aβ deposits towards the outer normal tissue.

• Component 3 (Fig. 3e and 4e) extends towards the outer layers of the plaque, hence it seems to be appropriate to identify it with the average elastic properties of the normal tissue (ECM). Its distribution denotes a second layer around the plaque core. Note that the scattering intensity gradually decreases from the core through to the periphery of the plaque owing to a decrease in thickness and/or density of the tissue.

• Component 4 (Fig. 3f and 4f) is the softest one, and is mainly localized in the region of the lipid ring,^4^,^14^ where an elevated (phospho)lipid signal has been associated with astrocyte processes (*i.e.* membrane extensions) radially projecting towards the plaque core. This is the component with the largest linewidth, suggesting more pronounced viscoelastic effects. This component can be attributed to the astrocytes and, given that the plasma membrane of astrocytes is mainly formed by lipids in these dry tissues, the stiffness measured here may essentially correspond to that of the lipid membrane hence providing a qualitative measure of the cell membrane's GHz mechanical properties.

Dispersion and absorption associated with the four components can be better seen in Fig. 5. A progressive reduction in frequency associated with an increase in linewidth is observed for the four components from #1 to #4, and this is consistent for the two plaques (red-to-blue symbols).

This plot reproduces the distinctive gradual change in physical properties going from the dense core through to the periphery of the plaque. A gradient of frequency shift and linewidth can be interpreted based on the distinctive structure of Aβ deposits whereby hydrophobic and hydrophilic compartments do cross over. The plaque core spectrum has a narrow peak around 18 GHz, whilst the intermediate regions show larger linewidths and depleted frequencies, and the astrocyte region has a linewidth that is approximately twice as large as the core and a frequency that is reduced by a quarter. From the frequency shift, the longitudinal elastic modulus at GHz frequency can be derived.^19^ This requires that the density and refractive index of the medium are known; generally an approximation is applied in that the ratio *ρ*/*n*^2^ is fairly constant far from electronic resonances.^14^ Here, by taking *n* = 1.36 (ref. 20) and *ρ* ∼ 1.0 g cm^–3^ and considering a 1% frequency shift due to the finite NA of the objective used, we obtained the elastic modulus for the distinct components of the plaque as being approximately 12 GPa (#1), 10 GPa (#2), 9 GPa (#3), and 8 GPa (#4). These trends suggest that the decrease in measured stiffness across the different components can also be promoted by a viscoelastic effect, *i.e.* a reduction of the relaxation time of molecular motions^21^ going from the core through to the periphery of the plaque.

## Discussion

Micro-Brillouin spectroscopy was used to characterize the micromechanics of hippocampal amyloid plaques in Aβ-overexpressing transgenic mice. The composition of such plaques is known to be rich in intermolecular β-sheet structures of the Aβ peptide, and dense-core plaques have a distinctive ‘halo’ encompassing the core that is formed by activated astrocyte processes.^4^ The change in mechanical properties across the Aβ deposits can be explained by a parallel change in the pattern of molecular species into the brain.^14^ Mice of two groups, transgenic (TASTPM) and normal control animals, were studied. A previous *in situ* examination of the same hippocampi has shown enhanced protein's IR absorbance and Raman scattering, indicating that plaques occurred in the TASTPM group.^4^ This was corroborated by correlative immunofluorescence imaging of the same hippocampi stained with amylo-glo. An important finding supported by medical literature^22^ was the detection of enhanced lipid signal in the region surrounding the plaque core that was attributed to astrocyte processes with the use of immunohistochemical techniques. Astrocytic activation associated with Aβ plaques has been known for some time, however this was the first demonstration that chemical imaging techniques can detect not only plaques but also associated astrogliosis, which might have a protective role limiting the development of amyloidosis-dependent cognitive decline.^23^

Brillouin microscopy and correlative micro-Raman analysis confirmed the heterogeneity in Aβ deposits through maps of Brillouin frequency shift (related to stiffness) and linewidth (related to apparent viscosity), which indicated that plaques have a rigid core rich in Aβ protein with a β-pleated sheet conformation, a viscoelastic lipid-rich layer surrounding the core, and an heterogeneous extracellular matrix plausibly disseminated of glial cell bodies.^14^ For these reasons, this micromechanical method may provide an additional contrast mechanism to detect amyloidopathy.

Here we used a new method based on non-negative matrix factorization (NMF)^17^ to decompose Brillouin hyperspectral datasets of Aβ plaques in the hippocampus of an Aβ-overexpressing transgenic mouse. In this method, the hyperspectral images are interpreted as a linear combination of a limited number of components, each characterised by a spectrum and a concentration map. We used as many components as required to describe most of the variance in the dataset, leaving only a randomly distributed factorisation error. We found that distinct spatial locations within Aβ plaques – which correspond to specific molecular species – have differential viscoelastic properties probed by Brillouin spectroscopy. Four components were required to describe the data, each characterised by a spectrum with a frequency and linewidth that change progressively when going from the core through to the periphery of the plaque. Component 1, the one with the largest rigidity, corresponds to the dense core made of aggregated Aβ peptide; component 2 can be identified as a transition region, plausibly including α-helical structures of the Aβ peptide with different levels of aggregation; component 3, which extends towards the outer layers of the plaque, can be assigned to the normal tissue's ECM; component 4 is the softest one and is mainly consistent with the lipid-rich layer^14^ previously associated with astrocyte processes.^4^ Given that the plasma membrane of astrocytes is mainly formed by lipids in these dry tissues, the stiffness measured here may essentially correspond to that of the lipid membrane hence providing a qualitative measure of the cell membrane's stiffness. Both components 1 and 2 are essentially homogeneous components with some damping, and the others (3 and 4) have some inhomogeneity in stiffness which reflects a distribution in material's parameters.

This is an important result because distinct components of plaques may be obscured by overlapping spectral contributions, whilst the multivariate method that analyses both spectral and spatial distribution within a hyperspectral dataset can retrieve the constituent components. The result may have clinical implications. In fact, the mechanical properties of the plaques may influence the interaction between the biologically active, soluble components of the plaque (Aβ monomers, oligomers, protofibrils and fibrils) and the surrounding tissues and cell types, including neurons, astrocytes, microglia and endocytes. This, in turn, may affect how the disease progresses. In addition, newly developed Aβ scavenging drugs aim at treating AD by removing pathogenic amyloid species from the brain parenchyma. Recent clinical trials of these compounds resulted in major setbacks,^24^,^25^ pushing the AD research community to reconsider the approach towards amyloidopathy, highlighting the need to better describe Aβ plaque complex chemistry and physical properties. Our approach, aiming at correlatively describing the physical and chemical properties of the plaques, may have a critical role in providing information on the development of novel Aβ scavenger compounds and the assessment of their efficacy.

It is noteworthy that the finite number of components used in the factorization method gives a coarse grained picture of the otherwise gradually varying properties of the tissue, so that *e.g.* Component 2 reflects the average properties of the transition region. Also, the method applied here makes no assumption on the lineshape of the single components; in a further implementation of the algorithm, the single spectra could be constrained to a viscoelastic model such as DHO and a set of homogeneous components could be retrieved. We emphasize that NMF extracts components and their spatial concentrations in a global analysis, which is specifically useful if the material investigated consists of a mixture of a few components. It also allows to describe inhomogeneous distributions within the focal volume, which create overlapping modes. In the traditional pixel-wise fitting of the spectra with a DHO model, such a distribution of viscoelastic properties will return an average frequency, and an overestimated broadening. Using multiple DHO components in such a fit is possible, but tends to be unstable. Since the NMF analysis uses all spectra in the dataset at the same time to extract the components and spectra, it is robust in dealing with multiple components. This is the first time that a multivariate statistical model was applied to Brillouin microscopy images to decompose the hyperspectral datasets in multiple distinct components.

In these measurements, the noise is simply the square root of the intensity, and the signal-to-noise ratio is considerably better than that typically obtained with VIPA spectrometers, due to the high luminosity of the FP interferometer^9^ and the adopted integration time. Whilst we have demonstrated that recent developments in scanning FP interferometers enable sub-second time resolution,^9^ here we investigated fixed samples and chose to have a very good signal-to-noise ratio to enable a detailed spectral bandshape analysis. Multivariate statistics can be used to analyse hyperspectral datasets acquired at a lower signal-to-noise ratio (hence faster acquisitions) and with suitably numerous datapoints. In this sense, even maps with larger point-to-point intervals can be exploited, hence speeding up the analysis. This has been shown using a combination of NMF and sparse sampling for CARS microscopy,^26^,^27^ and could be used also for Brillouin spectroscopy to allow for high speed mapping.

In summary, the results demonstrate the potential of Brillouin micro-spectroscopy for studying Alzheimer's disease. Specifically here, we have shown the application of multivariate analysis, which allowed the decomposition of the highly overlapped features forming the Brillouin spectrum of such complex specimens, and hence the recognition of different constituents. The results of NMF demonstrated structural motifs of the amyloid plaques that are difficult to separate *via* univariate analysis. In particular, NMF provided evidence of individual components of the tissues such as the dense core made of aggregated β-sheets of the Aβ peptide, transition regions plausibly characterised by α-helical structures and normal tissue, and astrocyte processes as the outermost layer. Thus, by taking into account spatial correlations among spectral features, multivariate analysis was able to discriminate between different components of the hippocampal CA1 region, while a univariate approach showed limitations. This demonstrated that the application of chemometric techniques allows information to be obtained from mechanically specific datasets, such as those measured by Brillouin micro-spectroscopy. Since Brillouin spectroscopy does not involve physical contact with, and hence no mechanical interference with, the specimen, it is ideally suited to human *in vivo* applications and, as it relies upon the propagation of longitudinal acoustic waves, it enables applications to tissues at depth. Brillouin microscopy with multivariate data analysis could evolve into an important tool for routine diagnosis of Alzheimer's disease if suitable *in vivo* probes become available. Analysis of the micromechanics of plaques might, for example, allow the identification of those likely to be ‘protected’ by astrogliosis and not cause clinical events.

## Methods

### Sample preparation

This study was carried out in accordance with UK Home Office Guidelines and the University of Exeter Animal Welfare Ethical Review Board. The protocol was approved by the University of Exeter Animal Welfare Ethical Review Board. Male 12 months old Aβ-overexpressing TASTPM (transgenic, TG) mice were used in this study. TASTPM mice carry two mutations on the APP gene (APP_Swe_ K670N, M671L) and one on the presenilin 1 gene (M146V) that can be found in patients affected by familial Alzheimer's disease.^15^,^16^ Animals were housed at room temperature under a 12 hour light cycle, and fed a normal diet with free access to food and water *ad libitum* before being sacrificed.^28^

Tissues were collected and sectioned as previously described^4^,^28^ before being mounted on Raman-grade polished calcium fluoride slides (Crystran, Poole, Dorset, UK). Two plaques within a brain hippocampal section of a TASTPM mouse were analysed, and two microspectroscopic maps were obtained.

### Brillouin microscopy

Brillouin micro-spectroscopy maps were collected with a customized JRS Scientific Instruments CM-1 confocal microscope equipped with a 532 nm laser and a Mitutoyo long working distance 20× (NA 0.42) objective. The backscattered light from the sample was dispersed through a (3 + 3)-pass Fabry–Perot interferometer onto a single photon avalanche photodiode detector.^9^ The spectral resolution was approximately 100 MHz. Characteristic Brillouin peaks due to the longitudinal acoustic wave propagation in the tissue were analysed, yielding maps with a spatial resolution in the range of 2 μm for the lateral and 8 μm for the axial dimension. These peaks are a proxy for the viscoelasticity of amyloid plaques.^14^ Brillouin maps were acquired in raster scanning mode with an exposure time of 90 s per point-spectrum and a step size of 1.5 μm, in the range –30 to 30 GHz, with 5 mW laser power on the sample. GHOST software was used for acquisition and manipulation of the data.

### Hyperspectral image analysis

For the factorisation analysis, we considered only the anti-Stokes part of the hyperspectral images for frequencies higher than ∼5 GHz, to suppress contributions from the Rayleigh scattering which would affect the analysis. In the case of the whole plaque image, first we removed the cosmic rays present in the spectra. Cosmic rays were identified as pixels where the intensity exceeded by five times the average intensity of the nearest neighbours. The hyperspectral images were then denoised by using a singular value decomposition (SVD) approach.^17^ In this method, noise reduction is achieved by applying SVD on the noise-whitened data and retaining only the decomposition components where both the left and right singular values do not show physically meaningful spectral and spatial features, respectively. For the plaque images, we considered four SVD components. Finally we analysed the denoised hyperspectral data by using NMF with four components. The method is unsupervised as random initial guesses for both spectra and concentration are used. To improve the reproducibility and limit the effect of the randomness of the guesses, we employed the knock-out method.^18^

### Fit analysis

The spectra derived from NMF applied to the Brillouin maps were fitted to a damped harmonic oscillator (DHO) function,^19^ arising from a viscoelastic model of homogeneous elastic properties.

## Author contributions

F. P. and F. T. conceived, designed and supervised the project. S. M. and D. F. performed experiments and viscoelastic analysis. F. M. and W. L. processed the data and performed the hyperspectral analysis. P. B., W. L., D. F. and F. T. helped with the discussion of the results. F. P. wrote the manuscript with input from all other authors.

## Conflicts of interest

The authors declare no competing financial interests.
